# Computational Exploration for Lead Compounds That Can Reverse the Nuclear Morphology in Progeria

**DOI:** 10.1155/2017/5270940

**Published:** 2017-10-26

**Authors:** Shailima Rampogu, Ayoung Baek, Minky Son, Amir Zeb, Chanin Park, Raj Kumar, Gihwan Lee, Donghwan Kim, Yeonuk Choi, Yeongrae Cho, Yohan Park, Seok Ju Park, Keun Woo Lee

**Affiliations:** ^1^Division of Applied Life Science (BK21 Plus), Plant Molecular Biology and Biotechnology Research Center (PMBBRC), Systems and Synthetic Agrobiotech Center (SSAC), Research Institute of Natural Science (RINS), Gyeongsang National University (GNU), 501 Jinju-daero, Jinju 52828, Republic of Korea; ^2^College of Pharmacy, Inje University, 197 Inje-ro, Gimhae, Gyeongnam 50834, Republic of Korea; ^3^Department of Internal Medicine, College of Medicine, Busan Paik Hospital, Inje University, Gyeongnam, Republic of Korea

## Abstract

Progeria is a rare genetic disorder characterized by premature aging that eventually leads to death and is noticed globally. Despite alarming conditions, this disease lacks effective medications; however, the farnesyltransferase inhibitors (FTIs) are a hope in the dark. Therefore, the objective of the present article is to identify new compounds from the databases employing pharmacophore based virtual screening. Utilizing nine training set compounds along with lonafarnib, a common feature pharmacophore was constructed consisting of four features. The validated Hypo1 was subsequently allowed to screen Maybridge, Chembridge, and Asinex databases to retrieve the novel lead candidates, which were then subjected to Lipinski's rule of 5 and ADMET for drug-like assessment. The obtained 3,372 compounds were forwarded to docking simulations and were manually examined for the key interactions with the crucial residues. Two compounds that have demonstrated a higher dock score than the reference compounds and showed interactions with the crucial residues were subjected to MD simulations and binding free energy calculations to assess the stability of docked conformation and to investigate the binding interactions in detail. Furthermore, this study suggests that the Hits may be more effective against progeria and further the DFT studies were executed to understand their orbital energies.

## 1. Introduction

Hutchinson-Gilford Progeria Syndrome, Progeria, is a rare genetic disorder seen in children and is manifested by premature aging [1]. This fatal disorder was studies by two scientists Jonathan Hutchison in the year 1886 and Hastings Gilford [2] in 1897 and hence it was named Hutchinson-Gilford Progeria Syndrome (HGPS) [3, 4]. Progeria is originated from the Greek terminology “progeros” that refers to “prematurely old” [5, 6] and affects 1 in 4–8 million [7, 8]. This syndrome can be observed in both sexes with 2 : 1 male/female ratio and across different countries showing no geographic and ethnic bias [8, 9]. The general life expectancy is approximately an average of 13 years and the affected may die due to several reasons [10]. Conversely, only a single case of a patient who lived 45 years of age exists [11]. The affected demonstrates a characteristic features by displaying delayed growth, osteoporosis, cardiovascular ailments, alopecia, pinched nose, and sclerodermatous skin [12, 13]. However, they display no change in the mental ability [4]. This is because the brain largely synthesizes lamin C and very little prelamin A [14].

Progeria is defined as sporadic autosomal dominant mutation [15], whose progression begins in utero [16]. Though the circumoral pallor symptom was found associated with the child at the time of birth, the delay in the phenomenal representation of the disease is due to the low levels of progerin during the undifferentiated embryonic cells and is demonstrated after the levels are elevated [17, 18].

The nuclear morphology is imperative in demonstrating progeria [19]. The abnormality of the nucleus is due to the mutations of two genes lamin (LMNA) and ZMPSTE 24 [20]. More specifically, the point mutations that occur in the lamin A/C are vital in demonstrating the disease [19, 21]. Dominant negative form of lamin A protein is produced as a consequence of the mutation G608G (GGC to GGT) within the exon 11 of lamin [22–24]. This mutation results in the formation of cryptic splice site subsequently causing the cleavage of 50 amino acid residues in the C-terminus of lamin A [19, 25, 26] and thus forms a protein named* progerin* with a distorted nucleus. This results in the deletion of site ZMPSTE24, demonstrating a permanent farnesylated protein leading to abnormal nucleus. Such cells with abnormal nucleus are prone to develop several diseases which are collectively referred to as laminopathies [27, 28] such as Emery-Dreifuss muscular dystrophy [29, 30], Dunnigan-type familial partial lipodystrophy [31, 32], and mandibuloacral dysplasia [33, 34]. It is therefore evident that the defective lamin A influences the instability of the protein thereby developing the diseases. Additionally, the accumulated progerin renders abnormalities in the behaviour of chromosome segregation and the reassembly of the nuclear envelop [16, 35]. Additionally, it dislocates the centromere protein-F (CENP-F) from kinetochores [16]. Subsequently, the genetic instability is elevated, thus favouring premature aging.

Despite alarming condition, promising therapeutic treatments are still under trails. Under such circumstances, drugs that were originally developed for certain diseases have proven to be effective against progeria [4]. Pravastatin, originally developed against cardiovascular diseases [36–38], zoledronic acid, a bisphosphate employed for treating osteoporosis [36–38], and farnesyltransferase inhibitors (FTIs) [36–38], used to treat cancers, have improved the condition of the progeroid children. Among them, the FTIs have ameliorated the diagnostic conditions in the affected by reverting the abnormalities of the nucleus [39–42]. Additionally, they have effectively improved the nuclear blebbing in the mouse models [43–46]. Mechanistically, FTIs operate by inhibiting the conversion of prelamin A to mature lamin A [13, 16, 47–49] and further improve the cardiovascular and skeletal pathologies besides gaining weight [4, 49]. Lonafarnib, one of the FTIs that has gained increasing popularity for treating progeria, has reached the clinical trials [16, 50, 51]. Owing to the beneficial effects of FTIs, it is mightily essential to identify new drugs which can perform with similar strategy. Therefore, in the current study, we focused on screening new chemical compounds that might be able to treat progeria using ligand-based pharmacophore method. In order to sieve the potential candidate compounds, the chemical features of lonafarnib have also been considered.

## 2. Materials and Methods

### 2.1. Generation of the Pharmacophore Model

**Figure 1:**
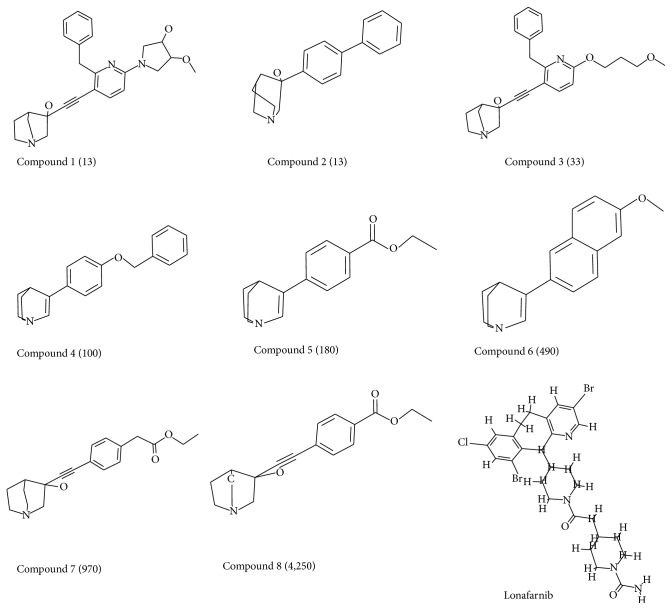
Training set compounds employed to build the pharmacophore. The chemical features of lonafarnib are also exploited in its construction. IC_50_ values are indicated in parentheses.

For the generation of the pharmacophore nine inhibitors, Figure 1, from various literatures have been considered along with lonafarnib with the known inhibitory activities [52, 53]. These inhibitors have displayed different IC_50_ values and diverse structure. The* common feature pharmacophore generation* protocol available on the Discovery Studio (DS) v4.5 (Accelrys, San Diego, CA) was used maintaining a minimum interfeature distance of 2.00 with* fast flexible* conformation generation. Common feature pharmacophore is generated using the* HipHop* algorithm that determines the common chemical features associated with the 3D spatial arrangements in a given training set. Additionally, the identification of the configurations is an exhaustive process that begins with a small set of features and proceeds on larger configurations. Subsequently, the pharmacophores are ranked as they are built, a method employed to explain the ability to map onto the pharmacophores and further their rarity. From the generated pharmacophores the best model was selected based upon the rank scores [54].

### 2.2. Validation of the Pharmacophore Model

The generated pharmacophore was validated adapting the Receiver Operating Characteristic (ROC) and the decoy set methods to assess its robustness in identifying the lead molecules. The ROC was initiated simultaneously during the generation of the pharmacophore. The nine inhibitors that were used to generate the pharmacophore model were taken as known actives and six other compounds were recruited as known inactives. On the other hand, the decoy set method was evaluated constituting a database of 107 compounds comprised of 9 active molecules. The quality of the pharmacophore was evaluated by computing the goodness of fit (GF) [55] score employing the formulae GH = {[Ha*∗*(3*A* + Ht)]/(4Ht*A*)}*∗*[1 − (Ht − Ha)/(*D* − *A*)] and EF = (Ha/Ht)/(*A*/*D*).

### 2.3. Database Screening for Retrieving the Virtual Candidates and Assessment of Drug-Like Properties

To identify the candidate drug compounds with high or similar potentiality, the validated pharmacophore has been used as a 3D query to search the chemical databases. Three databases have been used, namely, the Chembridge, Maybridge, and Asinex consisting of 50,000, 59,652, and 21,3262 compounds, respectively. Employing the* Ligand Pharmacophore Mapping* protocol implemented on the DS, the compounds were retrieved and were further culled setting the fit value as 3. Subsequently, obtained compounds were filtered on the bases of Lipinski's rule of five [56] and ADMET properties [57]. Lipinski's rule of five logically indicates that the well-absorbed compounds exhibits a log⁡*P* of less than 5, less than 5 hydrogen bond donors, and less than 500 molecular weight, respectively. Additionally, they also have the hydrogen bond accepting capability of less than 10. Furthermore, the compounds were checked by the ADMET descriptors to evaluate if they can cross the bold-brain barrier (BBB), possess low toxicity, and have good solubility and human intestinal absorption. Among them the key descriptors are the BBB and displaying no hepatotoxicity. The absorption, solubility, and the BBB were fixed at 3, 3, and 0, respectively [58, 59]. The resultant compounds were escalated to molecular docking along with the 9 compounds used for the generation of the pharmacophore.

### 2.4. Molecular Docking Mechanism

Molecular docking is one of the superior methods employed as a sampling method to identify the most accurate conformation [60]. Furthermore this technique also identifies the compounds that can fit into the active site of the target molecule and their corresponding interactions with the residues. For the current study, CDOCKER, implemented on the DS, was employed to understand the binding affinities of the protein and the ligand. The results were evaluated based upon the CDOCKER energy, while the CDOCKER interaction energy is used as a rescore. Highest CDOCKER interaction energy implies greater favourable binding [61, 62]. The protein target for the present research is the farnesyltransferase of high resolution (1.8 Å) with the PBD code 1TN6 bound with the cocrystal imported form the protein data bank [63]. The heteroatoms were removed performing the* clean protein* available in the DS. The protein was further minimized employing the CHARMm force field. Furthermore the binding site was evaluated 15 Å around the cocrystal and the histidine tautomers were protonated to ND1H state as was observed in the crystal structure. The key residues located at the active site have been identified as His748, Arg791, Lys794, and Tyr800 [63]. Each ligand was allowed to generate 100 conformers and depending upon the scoring functions and molecular interactions between the protein and the ligand, the ideal dock pose was chosen.

### 2.5. Molecular Dynamics Simulation

To further affirm the potentiality of the selected compounds and to evaluate the dynamic behaviour of the prospective drug molecules in the binding site pocket, they were subjected to MD simulations along with the reference compounds using GROMACS 4.5.7, employing CHARMm27 force field [64]. MD simulations were executed to examine the binding stability of the identified lead compounds in comparison with the reference compound. Ligand topologies were generated using SwissParam [65]. Corresponding counter ions were added to neutralize the solvated TIP3P water model present in the dodecahedron box. Unwanted contacts from the initial structure were dislodged by performing the energy minimization, adapting the steepest descent algorithm which was followed by the NVT and NPT equilibration steps. During this process, the solvent molecules along with the counter ions were allowed to move restraining the protein backbone. Both the processes were executed for 100 ps at 300 K and a pressure of 1 bar, respectively. Parrinello-Rahman barostat was employed to maintain the pressure of the system [66]. The geometry of the water molecules and the bonds involving hydrogen atoms were constrained employing the SETTLE [67] and LINCS [68], respectively. Particle Mesh Ewald (PME) [69] was used to calculate long-range electrostatic interactions. A cut-off distance of 12 Å was attributed for Coulombic and van der Waals interactions. The equilibrated structures were then subjected to production run which was conducted for 20 ns and the results were evaluated using VMD [70] and DS.

### 2.6. Binding Free Energy Calculations

To delineate further on the protein-ligand complex, time-dependent binding free energy calculations were performed. Molecular Mechanics/Poisson-Boltzmann Surface Area (MM/PBSA) [71, 72] was used for its accomplishment and was performed after the MD simulations. The obtained Δ*G* should take into account the protein fluctuations and the ligand conformations, which therefore ensures proper positioning of the ligand within the binding pocket.

The binding free energy protein-ligand complex in solvent system is stated as

(1) $$\begin{array}{c} \Delta G_{binding}=G_{complex}-\left(\;G_{protein}+G_{ligand}\;\right). \end{array}$$

Herein, *G*_complex_ refers to the total free energy of the complex and *G*_protein_ and *G*_ligand_ indicate the separated protein and ligand in the solvent. Their free energies can be computed by

(2) $$\begin{array}{c} G_{X}=E_{MM}+G_{solvation}, \end{array}$$

where *X* can be a protein, ligand, or its complex. *E*_MM_ represents the average molecular mechanics potential energy in vacuum, while *G*_solvation_ interprets the free energy present in the solvation.

Additionally, molecular mechanics potential energy in vacuum can be evaluated by adapting the equation

(3) EMM=Ebonded+Enon-bonded=Ebonded+Evdw+Eelec.

*E*_bonded_ represents the bonded interactions, while the nonbonded interactions are denoted by *E*_non-bonded_. Δ*E*_bonded_ is generally regarded as zero [73].

The solvation free energy (*G*_solvation_) is expressed by the sum of electrostatic solvation free energy (*G*_polar_) and a polar solvation free energy (*G*_non-polar_) and is given as follows:

(4) $$\begin{array}{c} G_{solvation}=G_{polar}+G_{\text{non-polar}}. \end{array}$$

*G*_polar_ is computed recruiting the Poisson-Boltzmann (PB) equation [74] while *G*_non-polar_ is computed from the solvent-accessible surface area (SASA) and can be written as follows:

(5) $$\begin{array}{c} G_{\text{non-polar}}=\gamma SASA+b. \end{array}$$

Here, *γ* is the coefficient of the surface tension of the solvent, whereas *b* is its fitting parameter, whose values are 0.02267 kJ/mol/Å^2^ or 0.0054 kcal/mol/Å^2^ and 3.849 kJ/mol or 0.916 kcal/mol, respectively.

### 2.7. Density Functional Theory

The MD optimized conformations were further examined by density functional theory (DFT) analysis implemented with DS. With DFT we can calculate the Kohn-Sham orbital energies by means of the Highest Occupies Molecular Orbital and (HUMO) and Lowest Unoccupied Molecular Orbital (LUMO) [75, 76]. Moreover, the ionization potential (electron donor capacity) was demonstrated by the HOMO, while the electron affinity (electron acceptor) was represented by LUMO. In order to understand the energy transfer and further the stability of the small molecules within the binding site [58, 77], the MD optimized docked conformations were subjected to DFT. A lower energy gap between the molecules demonstrates that the molecules are highly reactive while the higher energy gap implies low reactivity [78–80]. The Dmol3 and Becke, three-parameter, Lee-Yang-Parr (B3LYP) [81, 82], using the DND basis set with self-consistent field (SCF) density convergence of 1.0*e* − 6, available on the DS, was employed for computing the DFT. Additionally, the DFT studies were carried out to evaluate the electronic properties of the obtained Hits and the reference compound.

## 3. Results and Discussion

### 3.1. Generation of the Pharmacophore Model

**Figure 2:**
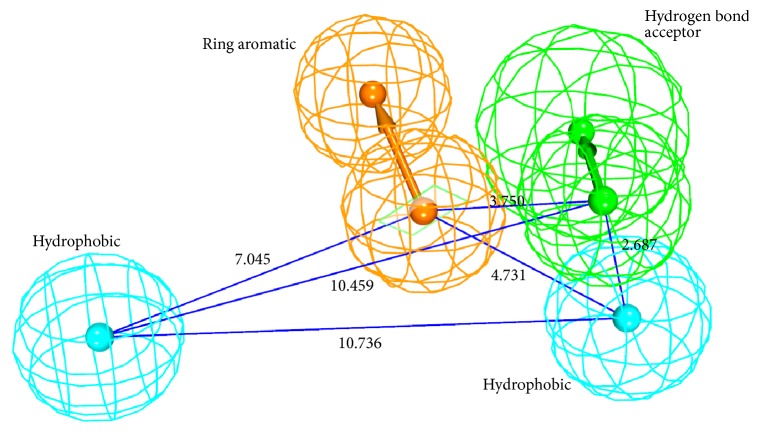
The best pharmacophore model (Hypo 1) consisting of four pharmacophoric features with its geometry.

Common feature pharmacophore module that utilizes the* HipHop* [83] algorithm implemented on the DS was employed to generate the pharmacophore with minimum features and maximum features being 1 and 10, respectively. Delineating on the pharmacophore features, it was observed that the HHDA feature was present in all the pharmacophores; however it was absent in one model. Furthermore, the ring aromatic (RA) feature was noticed in six models. It could therefore be understood that these features are the key features to be possessed by the drug molecules. Accordingly, care was taken in selecting an ideal pharmacophore that contains these features and hence Hypo 1 was selected as the best model displaying a rank score of 26.307, Table 1. Subsequently, a four-featured pharmacophore model was generated comprised of two-hydrophobic, one-ring aromatic, and one-hydrogen bond acceptor, Figure 2.

### 3.2. Validation of the Pharmacophore Model

**Figure 3:**
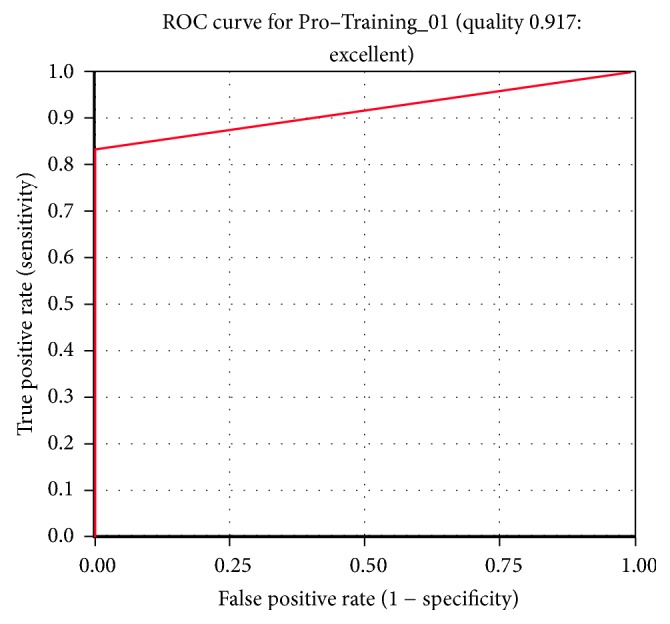
ROC curve. The quality of the pharmacophore was observed to be good.

The generated pharmacophore was validated utilizing two different methods, the ROC curve and the decoy set method. The ROC validation was performed during the pharmacophore generation. In this, the nine training set ligands are labeled as known actives and other nonspecific ligands are referred to as known inactives. Ideally, a good pharmacophore should recognize the known actives form the inactives and thus evaluates the quality of the pharmacophore. The generated pharmacophore was successful in identifying the known actives and displayed an excellent quality, Figure 3.

In the decoy set method, an external database of 107 compounds has been instituted consisting of nine actives (*A*).* Ligand pharmacophore mapping* available on DS was then launched. Pharmacophore has mapped with nine compounds (Ht) in which eight compounds were the active compounds (Ha) conferring 88.8% yield of actives. Furthermore, the (Goodness of Hit Score) GH and the (Enrichment Factor) EF scores have been computed to be 0.71 and 9.4, respectively. Generally, the GH score lies between 0 and 1, where 0 represents null model while 1 indicates the best model. Furthermore, good model should have a GH score above 0.7 [84, 85]. Accordingly, the generated Hypo1 is considered good as it demonstrated a score of 0.71. Different calculations computed as a part of decoy set validation are tabulated in Table 2.

### 3.3. Database Screening for Retrieving the Virtual Candidates and Assessment of Drug-Like Properties

**Figure 4:**
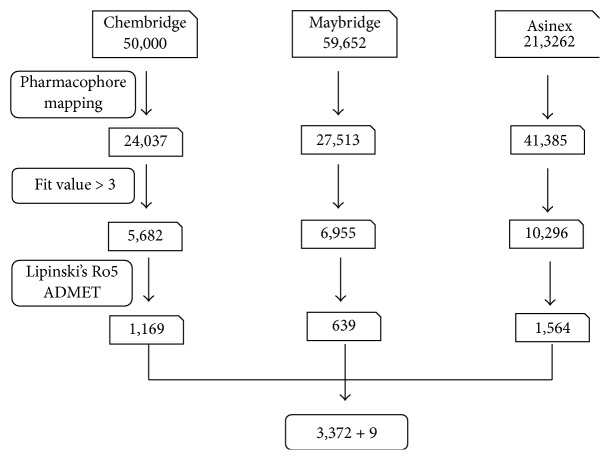
Different steps involved in screening the candidate compounds. 3,372 compounds are obtained from screening and 9 compounds belong to the training set.

Virtual screening is a process of determining every small molecule provided in the databases so as to judge their capability of binding with the target protein. Pharmacophore based virtual screening largely depends upon the chemical features present on the pharmacophore in critically assessing and identifying the candidate compounds from the databases. In the current study, the pharmacophore has mapped with 24037, 27513, and 41385 compounds of Chembridge, Maybridge, and Asinex databases, respectively. Subsequently, compounds were filtered based upon the fit value greater than five. As a result, the obtained 5682, 6955, and 10296 compounds were examined for their drug-like properties using the Lipinski's rule of 5 and ADMET. Consequently, a total of 3372 compounds were obtained, Figure 4, and were forwarded to molecular docking along with the training set compounds.

### 3.4. Molecular Docking Mechanism

**Figure 5:**
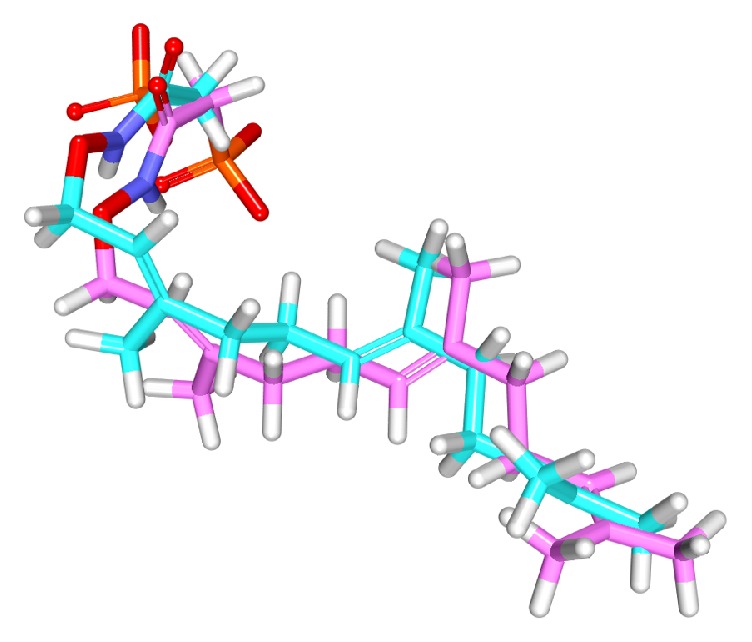
Validating the docking parameters using the cocrystal. Pink refers to the cocrystal and cyan refers to the docked pose.

**Figure 6:**
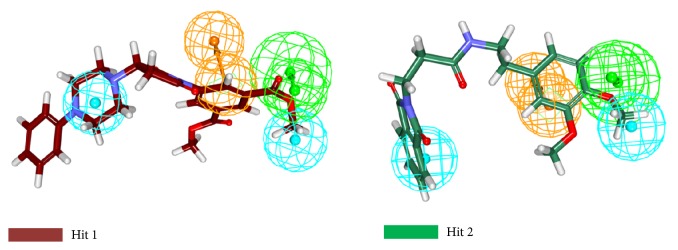
Mapping of the Hits onto the pharmacophore. The Hits are found to map with all the features of the pharmacophore.

The compounds obtained from the previous steps along with the nine compounds were subjected to molecular docking. Hereinafter, the compound with the lowest IC_50_ value from the nine training set compounds is referred to as the reference compound. In order to assess the suitability and accuracy of the CDOCKER prior to the commencement of the protocol, the cocrystal ligand was docked into the crystal structure of the target. The resultant pose has generated an acceptable RMSD of 1.4 Å, Figure 5. For assessing the dock results, the reference dock scores and the scores of lonafarnib were considered. The reference has generated an interaction energy of 23.5208 kcal/mol, while the lonafarnib has displayed 50.6141 kcal/mol, Table 3. In pursuit of identifying the most biologically active compounds, the compounds that have demonstrated higher CDOCKER interaction energy and higher CDOCKER energy than the reference and lonafarnib have been considered for further studies. Consequently, a total of six compounds have been retrieved from Asinex, Chembridge, and Maybridge databases. These compounds were further probed for their interactions with the residues that reside at the active site groove. Among the six Hit compounds listed in Table 3, only two compounds (CHEM, AXN_4) were observed to show interactions with the key residues and mapped with all the features exhibited by Hypo 1, Figure 6, and therefore these compounds were escalated to the MD simulations.

### 3.5. Molecular Dynamics Simulations

**Figure 7:**
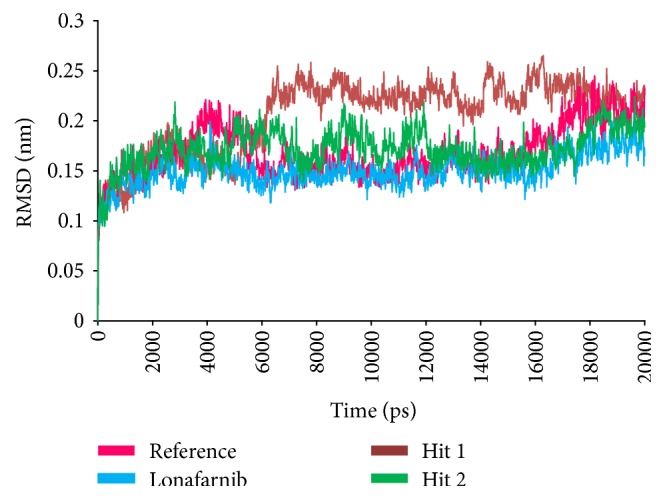
Structural stability analysis through RMSD of the four complexes.

**Figure 8:**
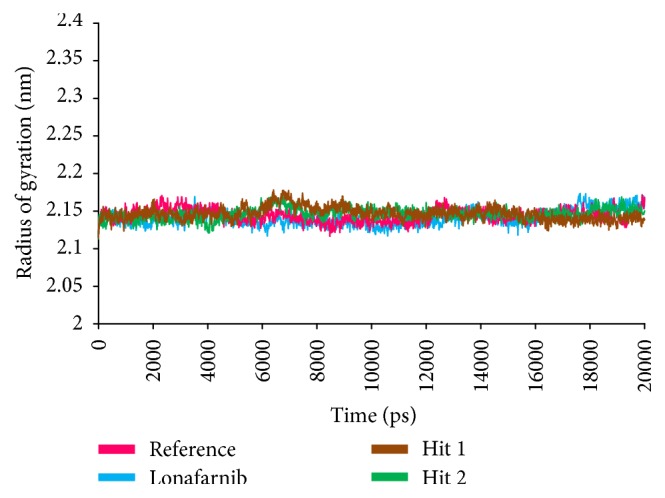
Radius of gyrations of the four systems to understand their compactness.

To further investigate the structural stability and to evaluate the dynamic behaviour of the Hit compounds in the binding site of farnesyltransferase, the MD simulation was executed for the reference, lonafarnib, and the Hits. Accordingly, 20 ns run was initiated to understand the conformational variations and the nature of the ligand within the active site. For this assessment, the docked poses were determined as the initial conformations. Subsequently, the root mean square deviation (RMSD) of the protein backbone atoms and the radius of gyration (Rg) were evaluated. The RMSD of all the complexes was found to be within 0.27 nm; however, Hit 1 has displayed slightly higher RMSD of 0.25 nm, while the others were stable at 0.2 nm, Figure 7. Furthermore, it was noticed that towards the last 3 ns the systems were converged. Additionally, the radius of gyration that implies the compactness of the proteins, revealed that the four systems are finely folded with no major aberrations after 8000 ps and are represented between 2.13 and 2.15 nm, Figure 8.

**Figure 9:**
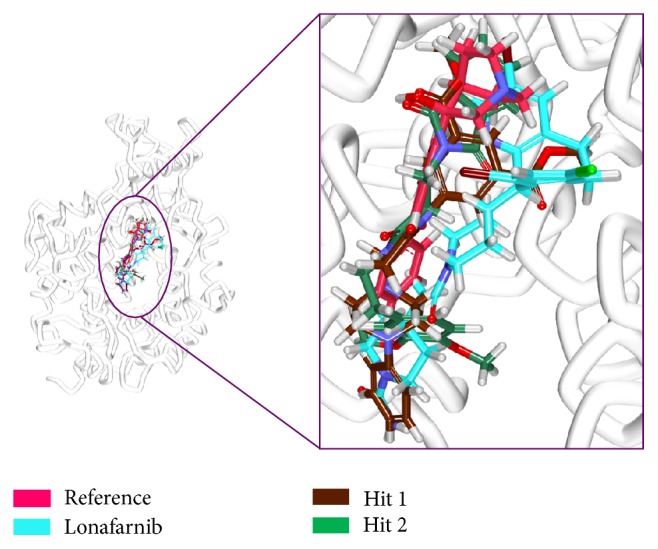
Binding mode analysis of the reference the Hits. Picture on the left represents the superimposed structure and on the right is its enlarged form.

**Figure 10:**
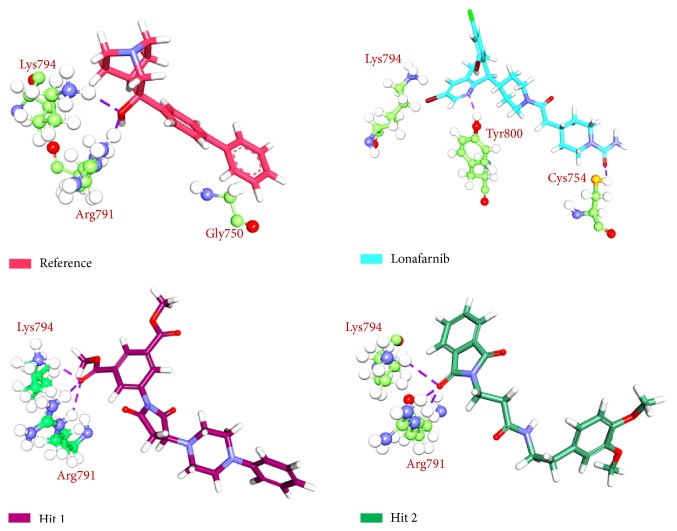
Hydrogen bond interactions between the protein and the ligands. Purple dashed lines denote the hydrogen bonds.

**Figure 11:**
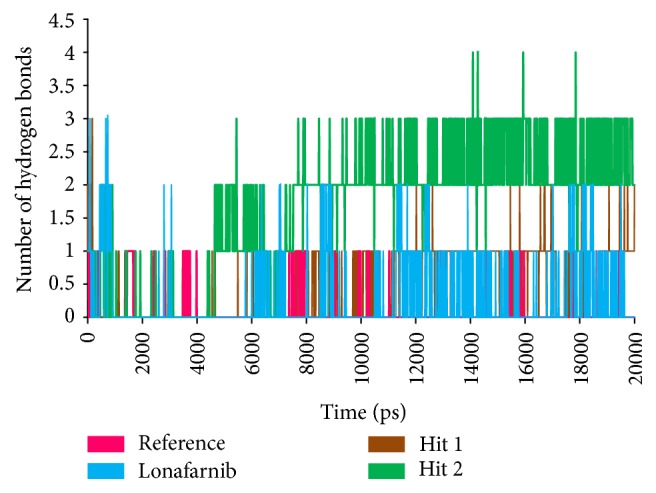
The number of intermolecular hydrogen bonds between protein and the ligands during 20 ns MD simulations.

**Figure 12:**
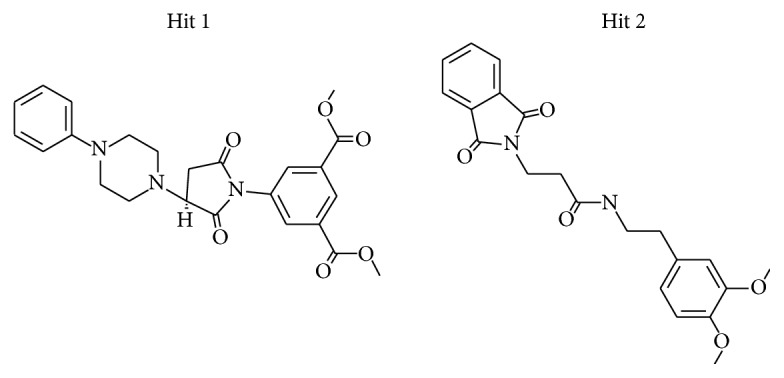
2D structures of the Hit compounds.

To further analyse the binding modes of each compound, the representative structures of the last 3 nm were obtained as it was observed to be well converged, Figure 7. Upon superimposition of the corresponding structures, it was established that the binding fashion of the Hits was in agreement with lonafarnib and the reference compound, Figure 9. The Hits were thereafter analysed for the intermolecular interactions with the crucial residues. Reference compound has formed two hydrogen bonds with Arg 791 and Lys794 with a distance of 2.2 Å and 1.9 Å, respectively. Lonafarnib has demonstrated two hydrogen bonds through Cys754 and Tyr800 represented by a length of 2.2 Å and 2.1 Å. On the contrary, the Hits have demonstrated three hydrogen bonds each. Hit 1 has produced two hydrogen bonds with Arg791 and Lys794, represented by a bond length of 2.4 Å, 2.0 Å, and 2.0 Å, respectively. Similarly, Hit 2 also has displayed three hydrogen bonds, one with Lys794 and two with Arg791, portraying a length of 2.6, 1.8, and 1.9, correspondingly, Figure 10. Additionally, the benzene ring of the reference has formed the *π* interaction while Tyr751 and Trp803 have been involved in *π*-*π* T-shaped interactions with the other benzene ring. Lonafarnib has formed a *π*-anion bond with Asp797. Additionally, the Br group of the ligand has interacted with Alkyl with Leu795. Furthermore, the benzene ring has formed *π*-alkyl bond with Arg702 and Trp803, respectively. The two extreme benzene rings Hit 1 have displayed *π*-*π* stacked bonds with Tyr800 and Trp803 residues. Additionally, the Arg702, Tyr751, Cys799, and His862 have formed the alkyl and *π*-alkyl bonds. Hit 2 on the contrary has projected *π*-*π* stacked bonds with Tyr800. Upon closer look at the interactions, it is evident that Hit 1 has showed greater number of bonds which may imply its higher efficiency. The details of the interactions are tabulated in Table 4 and Supplementary 1 in Supplementary Material available online at https://doi.org/10.1155/2017/5270940. Moreover, to delineate on the hydrogen bonds and to gain insight and understand the nature of the ligand in the active site, the hydrogen bond interactions were monitored throughout the MD run. The Hits have demonstrated greater hydrogen bonds as compared to the reference and lonafarnib, displaying average hydrogen bonds of 0.4 and 1.6, respectively. The reference has shown 0.07 and lonafarnib has projected 0.2 hydrogen bonds at an average. Furthermore, the reference has rendered marginal hydrogen bonds between 12000 to 15000 ps. On the contrary, the Hits have demonstrated regular hydrogen bonds during the MD run, Figure 11. Additionally, their 2D structures are depicted in Figure 12, Hit 1 was retrieved from Chembridge database, and Hit 2 was obtained from Asinex database. Furthermore, probing the PubChem online search tool, it was affirmed that these compounds have not been explored against progeria.

### 3.6. Binding Free Energy Calculations

**Figure 13:**
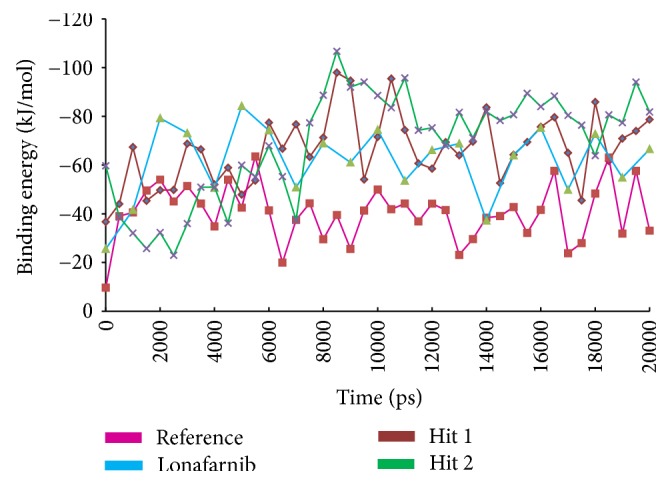
Estimation of binding free energy using MM/PBSA approach conducted during 20 ns.

**Figure 14:**
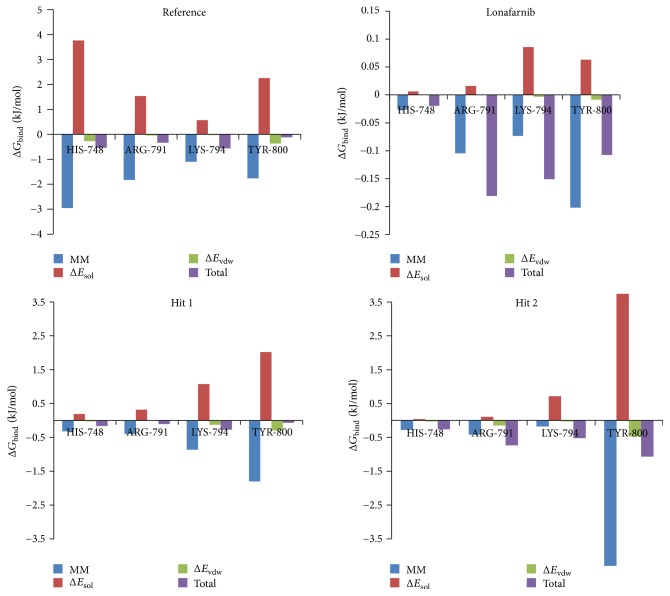
Perresidue decomposition of the binding energies of the key residues.

MM/PBSA method was employed to compute the binding free energies for a given set of protein-ligand complex. In order to carry out this study, 20 snapshots [86] were evenly extracted for the four systems. These systems have displayed a Δ*G* between −20 kJ/mol~−110 kJ/mol; however, slight variations were noticed because the conformational space was not sampled enough. Additionally, the reference and lonafarnib have demonstrated a −32.78 kJ/mol and −60.39 kJ/mol, respectively. The Hits on the other hand were conferred with −64.38 kJ/mol (Hit 1) and −65.74 kJ/mol (Hit 2) demonstrating much lower binding energies than the reference and the lonafarnib, Figure 13. The generated binding energies are the sum of ligand conformations and the protein fluctuations so as to affirm proper positioning of the ligand in the active site cavity. Accordingly, the ligands (Hits) are seated in the charged active site of the farnesyltransferase through the hydrogen bond and the van der Waals interactions. Additionally, various hydrophobic interactions such as *π*-*π*, *π*-*π* T-stalked and *π*-alkyl bonds hold the ligand firmly within the active site. Furthermore, the Hits have displayed higher CDOCKER interaction energy and lower binding free energies, thus making themselves valuable in treating progeria. Delineating on the perresidue energy contribution, it was evident that in Hit 1 and Hit 2 Lys794 and Tyr800 have contributed majorly to the total energy as was seen with lonafarnib. Furthermore, His748 in the reference compound was a major contributor; however, in the other ligands the same impact was not displayed. Across all the ligands, Tyr800 was the largest contributor of the respective energy terms. Therefore, it can be deduced that Try800 might be imperative in inducing the inhibitory activity, Figure 14.

### 3.7. Density Functional Theory

The molecular orbital energies were calculated in order to assess the HOMO and LUMO that are responsible for the charge transfers in a given chemical reaction and further describes a molecule to be encountered by the electrophiles and nucleophiles, respectively. Additionally, the band gap generated between the HOMO and LUMO demonstrates the reactivity of the molecules corresponding to smaller gap being more reactive and wider gap implies less reactive, Table 5, and therefore, the molecules with smaller band gap are considered. For the current study, the MD optimized lead conformations of the ligands were forwarded to the HOMO and LUMO analysis. The Hit molecules were chosen based upon the least energy gap as compared to the reference compound. Additionally, the electrostatic potential maps were computed to probe into the structural aspects of a molecule as it significantly plays a key role in the receptor-ligand interactions and are computed for a set of points in the molecule [87]. The electrostatic potential maps of the corresponding compounds have been obtained by the superimposition of the electrostatic potentials upon the electron density surfaces of the compound [88]. Furthermore, the electron density is plotted by the intensity of the colour that reflects the characteristic feature of a molecule. Subsequently, the red colour refers to the high negatively charged region and thus corresponds to high charge accumulation and the blue represents the charge depletion [89] and is positively charged region. The intermediate colours, such as orange, yellow, and green demonstrate the charges midway between both the extremes [90]. The order of the colour magnitude can be demonstrated as

  (Highly negative) red < orange < yellow < green < blue (highly positive).

**Figure 15:**
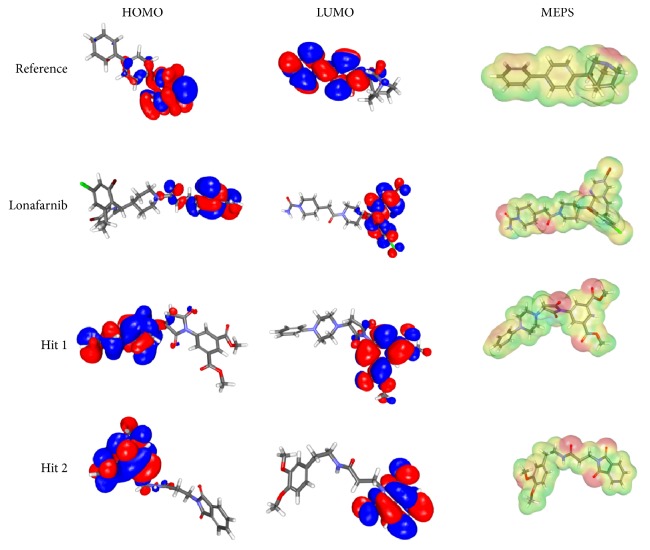
DFT studies for the MD optimized conformations. The HOMO, LUMO, and the molecular electrostatic profiles of the four systems.

Furthermore, the electrostatic potentials have been plotted to evaluate the electrostatic interactions that exists between the protein and the ligand. In the current study, the electron density isovalue was taken as 0.03 with a default colour scheme that ranged between 0.05 and 0.1. The positive phase of the molecular orbitals utilizes an isovalue of 0.01 and is demonstrated in blue, while the negative phase uses an isovalue of −0.01 and is depicted in red. The half-way potentials between them are represented by the other aforementioned colours. Furthermore, the molecular features that are resulted from the self-consistent field (SCF) corresponds to the electrostatic potential atomic-centered charges depicted by the molecular electrostatic potential (MEP) Figure 15 [91–93]. Additionally, MEP was calculated by a host of points determined on the 3D structures. Additionally, the electrostatic potential charges can be used to compute the intermolecular properties rather than the intramolecular properties. However, the buried atoms that are not on the outer region of the molecular van der Waals (vdW) surface are not identified as MEP points and are read by the lower relative root mean square (RRMS) values [94, 95]. The lower the RRMS, the higher the accuracy of the fit demonstrated by the MEP that in turn is calculated by QM produced by the fitted charges for the individual atoms [94, 95], Supplementary 2. As compared to the reference compound, the Hits have generated lower RRMS fit values and are relatively equal to the lonafarnib, Supplementary 2. Furthermore, the Mulliken atomic charges [96] were computed with spin 0 for the MD optimized ligands, specifically to those atoms that are involved in the hydrogen bonds and are tabulated in Table 6. These charges are related to the overlap matrix of the atomic orbitals [97]. Mulliken atomic charges of oxygen atoms have ranged between −0.44 and −0.52 au, while the nitrogen atom has displayed relatively lower charge of −0.303 au.

These results together with the HOMO and LUMO significantly portray the high electronegativity of the atoms. Furthermore, it can be observed that the Hits have demonstrated higher CDOCKER interaction energy and lower binding free energies and lower band gap as compared to the reference and lonafarnib. It can therefore be stated that the identified Hits have a similar efficacy or better electronic properties than the reference compounds in treating progeria.

## 4. Conclusion

Progeria is one of the rare genetic disorders manifested by premature aging in children leading to death. However, the currently available drugs are limited to clinical trials and therefore there is an essentiality for the discovery of new lead compounds. In the present article, we have successfully evaluated the potentiality of the novel lead candidates from the pharmacophore modelling coupled with molecular modelling studies. Furthermore, the putative binding modes of Hits have been examined upon comparison with the reference compound and the lonafarnib. Overall the results obtained from molecular docking, MD simulations, binding free energy, and DFT calculations indicate that the Hits have shown similar binding patterns with the reference and lonafarnib and have rendered strong molecular interactions with the active site residues of farnesyltransferase, thus qualifying as substantial compounds of interest in treating the Hutchinson-Gilford Progeria Syndrome.

## Supplementary Material

Supplementary1: Detailed 2D depiction of the interactions.
Supplementary2: Details of the ESP fitting calculations.

## Figures and Tables

**Table 1:** Common feature pharmacophore results as generated by the *HipHop*.

Hypo number	Features	Rank	Direct Hit	Partial Hit	Max fit
1	RHHDA	26.307	1111	0000	4
2	RHHDA	25.624	1111	0000	4
3	ZHHDA	25.574	1111	0000	4
4	RHHDA	25.352	1111	0000	4
5	RHHDA	25.352	1111	0000	4
6	HHHDA	24.569	1111	0000	4
7	RHHDA	24.450	1111	0000	4
8	HHHDA	24.362	1111	0000	4
9	ZHHDA	24.290	1111	0000	4
10	RHAA	24.069	1111	0000	4

R: ring aromatic, H: hydrophobic, HD: hydrogen bond donor, A: hydrogen bond acceptor, Z: zinc binder.

**Table 2:** Different parameters computed by decoy set method. The GF score confirms the predictive ability of the pharmacophore.

S. number	Parameters	Values
(1)	Total number of molecules in database (*D*)	107
(2)	Total number of actives in database (*A*)	9
(3)	Total number of Hit molecules from the database (Ht)	9
(4)	Total number of active molecules in Hit list (Ha)	8
(5)	% yield of active (Ha/Ht)	88.8
(6)	% ratio of actives [(Ha/*A*) × 100]	88
(7)	Enrichment factor (EF)	9.4
(8)	False negatives (*A* − Ha)	1
(9)	False positives (Ht − Ha)	1
(10)	Goodness of fit score (GF)	0.71

GH = {[Ha*∗*(3*A* + Ht)]/(4Ht*A*)}*∗*[1 − (Ht − Ha)/(*D* − *A*)] and EF = (Ha/Ht)/(*A*/*D*).

**Table 3:** Docking results according to CDOCKER interaction energy of the potential candidates. Dock scores higher than reference and lonafarnib were considered.

S. number	Name of the compound	-CDOCKER energy (kcal/mol)	-CDOCKER interaction energy (kcal/mol)
(1)	AXN_1	23.97	56.60
(2)	AXN_2	20.47	51.07
(3)	AXN_3	20.51	51.67
(4)	AXN_4	24.13	52.01
(5)	CHEM	28.53	51.33
(6)	MAY	40.37	52.38
(7)	Lonafarnib	20.16	50.61
(8)	Reference	22.57	23.52

**Table 4:** Different interactions rendered between the protein and the ligands.

S. number	Name of the compound	Hydrogen bonds < 3 Å				*π*-bonds	Van der Waals interactions
		Residue	atom	Ligand atom	Bond length		
(1)	Reference	Lys794 Arg791	HZ2 HH21	O21 O21	2.2 1.9	His748 Tyr751 Trp803	Leu795, Asp797, Lys853, Gly790, Tyr861, Gly750, Arg702, Tyr800

(2)	Lonafarnib	Cys754 Tyr800	HG HH	O5 N8	2.2 2.1	Arg702, Asp797, Leu795, Trp 803	Trp602, Tyr654, Tyr705, Cys706, His748, Gly750, Phe753, Arg791, Lys794, Val796, Asp852, Lys853

(3)	Hit 1	Arg791 Arg791 Lys794	HE HH21 HZ2	O24 O24 O24	2.4 2.0 2.0	Arg702, Tyr751, Cys754, Tyr800 His862	Trp602, Ala651, Tyr705, Cys706, Tyr 705, His748, Gly790, Leu795, Asp797, Cys799, Asp852, Lys856, Tyr861

(4)	Hit 2	Lys794 Arg791 Arg791	HZ2 HH21 HE	O10 O10 O10	2.6 1.8 1.9	Tyr800	Trp606, Tyr751, Gly750, Cys754, Leu795, Val796, Asp797, Trp803, Asp852, Lys853, Trp803, Tyr861, Tyr865

**Table 5:** HOMO, LUMO, and band gap of the Hits and the reference compounds computed employing the DFT approach.

Name	HOMO (eV)	LUMO (eV)	Band gap (eV)
Reference	−0.163	−0.0547	0.108
Hit 1	−0.154	−0.095	0.058
Hit 2	−0.179	−0.1149	0.064
Lonafarnib	−0.182	−0.0766	0.105

**Table 6:** Mulliken atomic charges of the atoms involved in hydrogen bonds.

Compound name	Ligand atoms	Mulliken atomic charges (au)
Reference	O2	−0.449
Lonafarnib	O5	−0.527
	N8	−0.303
Hit 1	O24	−0.448
Hit 2	O10	−0.437
