# Supplementary material for: Computational Exploration for Lead Compounds That Can Reverse the Nuclear Morphology in Progeria
Source: Biomed Res Int. 2017 Oct 26;2017:5270940. doi: 10.1155/2017/5270940 (PMC5684607; doi:10.1155/2017/5270940)
Supplement: Supplementary file 1 — Supplementary1: Detailed 2D depiction of the interactions. Supplementary2: Details of the ESP fitting calculations. [file 5270940.f1.zip › Supplementary 1.docx]

Supplementary 1 :2D depiction of the interactions


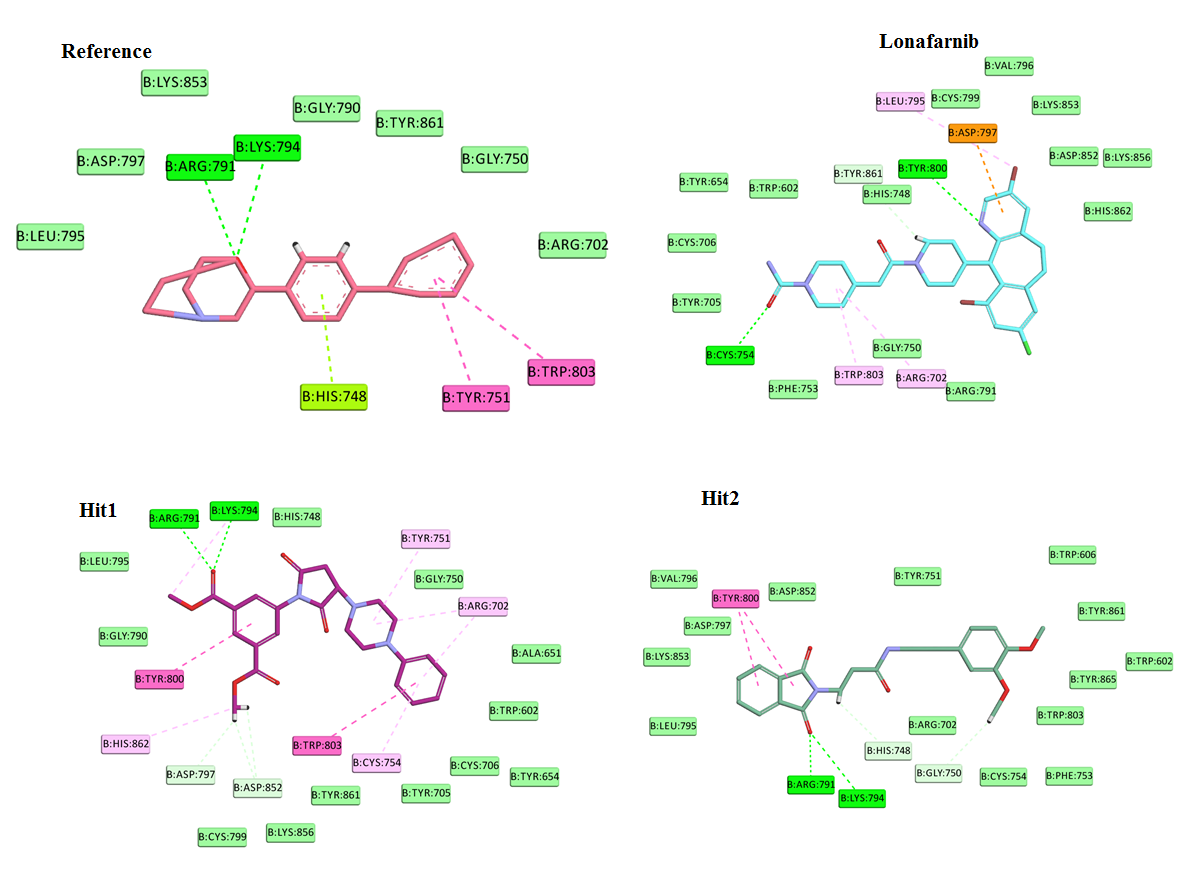


2D representation of the binding of the ligands to various active residues of the protein.
