# Supplementary material for: Computational Exploration for Lead Compounds That Can Reverse the Nuclear Morphology in Progeria
Source: Biomed Res Int. 2017 Oct 26;2017:5270940. doi: 10.1155/2017/5270940 (PMC5684607; doi:10.1155/2017/5270940)
Supplement: Supplementary file 1 — Supplementary1: Detailed 2D depiction of the interactions. Supplementary2: Details of the ESP fitting calculations. [file 5270940.f1.zip › Supplementary 2.docx]

**REFERENCE**

Summary of ESP fitting calculations

Number of points : 182461

Total integration weight : 41230.69

Spacing between : 0.25 Ang

Sigma : 0.1387E-02

RMS of V(exact) : 0.9923E-02

RRMS fit : 13.98 %

ESP-fitted charges :

n Elem chg vdW(in) vdW(ex)

1 C -0.015 2.00 3.50

2 C -0.209 2.00 3.50

3 C -0.073 2.00 3.50

4 C -0.171 2.00 3.50

5 C -0.120 2.00 3.50

6 C -0.025 2.00 3.50

7 C 0.123 2.00 3.50

8 C -0.159 2.00 3.50

9 C -0.123 2.00 3.50

10 C -0.135 2.00 3.50

11 C -0.113 2.00 3.50

12 C -0.174 2.00 3.50

13 C -0.152 2.00 3.50

14 C -0.083 2.00 3.50

15 N -0.524 1.83 3.33

16 C -0.007 2.00 3.50

17 C 0.453 2.00 3.50

18 C 0.160 2.00 3.50

19 C -0.019 2.00 3.50

20 C -0.193 2.00 3.50

21 O -0.635 1.72 3.22

22 H 0.056 1.30 2.80

23 H 0.032 1.30 2.80

24 H 0.103 1.30 2.80

25 H 0.117 1.30 2.80

26 H 0.083 1.30 2.80

27 H 0.105 1.30 2.80

28 H -0.038 1.30 2.80

29 H 0.103 1.30 2.80

30 H 0.093 1.30 2.80

31 H 0.053 1.30 2.80

32 H 0.067 1.30 2.80

33 H 0.365 1.30 2.80

34 H 0.107 1.30 2.80

35 H 0.113 1.30 2.80

36 H 0.109 1.30 2.80

37 H 0.114 1.30 2.80

38 H 0.117 1.30 2.80

39 H 0.124 1.30 2.80

40 H 0.126 1.30 2.80

41 H 0.123 1.30 2.80

42 H 0.121 1.30 2.80

**Hit1**

Summary of ESP fitting calculations

Number of points : 256841

Total integration weight : 55376.99

Spacing between : 0.25 Ang

Sigma : 0.1329E-02

RMS of V(exact) : 0.1563E-01

RRMS fit : 8.50 %

ESP-fitted charges :

n Elem chg vdW(in) vdW(ex)

1 N -0.052 1.83 3.33

2 C 0.447 2.00 3.50

3 C 0.344 2.00 3.50

4 C -0.132 2.00 3.50

5 C 0.043 2.00 3.50

6 C -0.030 2.00 3.50

7 C -0.272 2.00 3.50

8 C 0.653 2.00 3.50

9 C 0.233 2.00 3.50

10 O -0.443 1.72 3.22

11 O -0.466 1.72 3.22

12 C 0.223 2.00 3.50

13 C -0.263 2.00 3.50

14 O -0.536 1.72 3.22

15 N -0.610 1.83 3.33

16 C -0.197 2.00 3.50

17 C 0.190 2.00 3.50

18 C -0.365 2.00 3.50

19 O -0.283 1.72 3.22

20 C -0.035 2.00 3.50

21 C -0.119 2.00 3.50

22 O -0.314 1.72 3.22

23 C 0.110 2.00 3.50

24 C -0.319 2.00 3.50

25 C -0.115 2.00 3.50

26 C -0.076 2.00 3.50

27 C -0.154 2.00 3.50

28 C -0.106 2.00 3.50

29 H 0.080 1.30 2.80

30 H 0.094 1.30 2.80

31 H 0.115 1.30 2.80

32 H 0.075 1.30 2.80

33 H 0.128 1.30 2.80

34 H 0.342 1.30 2.80

35 H 0.159 1.30 2.80

36 H 0.191 1.30 2.80

37 H 0.116 1.30 2.80

38 H 0.133 1.30 2.80

39 H 0.083 1.30 2.80

40 H 0.059 1.30 2.80

41 H 0.145 1.30 2.80

42 H 0.100 1.30 2.80

43 H 0.123 1.30 2.80

44 H 0.079 1.30 2.80

45 H 0.085 1.30 2.80

46 H 0.074 1.30 2.80

47 H 0.115 1.30 2.80

48 H 0.068 1.30 2.80

49 H 0.143 1.30 2.80

50 H 0.138 1.30 2.80

**Hit2**

Summary of ESP fitting calculations

Number of points : 330105

Total integration weight : 62002.47

Spacing between : 0.25 Ang

Sigma : 0.1473E-02

RMS of V(exact) : 0.1519E-01

RRMS fit : 9.69 %

ESP-fitted charges :

n Elem chg vdW(in) vdW(ex)

1 N -0.039 1.83 3.33

2 C 0.380 2.00 3.50

3 C 0.452 2.00 3.50

4 C -0.084 2.00 3.50

5 C -0.387 2.00 3.50

6 C 0.318 2.00 3.50

7 N -0.265 1.83 3.33

8 N -0.019 1.83 3.33

9 C 0.004 2.00 3.50

10 C 0.022 2.00 3.50

11 C 0.656 2.00 3.50

12 C 0.643 2.00 3.50

13 C -0.315 2.00 3.50

14 C -0.281 2.00 3.50

15 C -0.196 2.00 3.50

16 O -0.436 1.72 3.22

17 O -0.468 1.72 3.22

18 C -0.052 2.00 3.50

19 C -0.093 2.00 3.50

20 C -0.267 2.00 3.50

21 C -0.199 2.00 3.50

22 C 0.228 2.00 3.50

23 O -0.506 1.72 3.22

24 O -0.511 1.72 3.22

25 O -0.236 1.72 3.22

26 O -0.227 1.72 3.22

27 C -0.262 2.00 3.50

28 C -0.247 2.00 3.50

29 C -0.291 2.00 3.50

30 C -0.240 2.00 3.50

31 C -0.081 2.00 3.50

32 C -0.071 2.00 3.50

33 C -0.200 2.00 3.50

34 H 0.153 1.30 2.80

35 H 0.155 1.30 2.80

36 H 0.171 1.30 2.80

37 H 0.176 1.30 2.80

38 H 0.162 1.30 2.80

39 H 0.094 1.30 2.80

40 H 0.079 1.30 2.80

41 H 0.112 1.30 2.80

42 H 0.143 1.30 2.80

43 H 0.072 1.30 2.80

44 H 0.112 1.30 2.80

45 H 0.131 1.30 2.80

46 H 0.118 1.30 2.80

47 H 0.107 1.30 2.80

48 H 0.132 1.30 2.80

49 H 0.129 1.30 2.80

50 H 0.146 1.30 2.80

51 H 0.149 1.30 2.80

52 H 0.153 1.30 2.80

53 H 0.140 1.30 2.80

54 H 0.133 1.30 2.80

55 H 0.137 1.30 2.80

56 H 0.120 1.30 2.80

57 H 0.117 1.30 2.80

58 H 0.130 1.30 2.80

**Lonafarnib**

Summary of ESP fitting calculations

Number of points : 468850

Total integration weight : 68466.59

Spacing between : 0.25 Ang

Sigma : 0.1311E-02

RMS of V(exact) : 0.1586E-01

RRMS fit : 8.27 %

ESP-fitted charges :

n Elem chg vdW(in) vdW(ex)

1 Br 0.059 2.16 3.66

2 Br 0.029 2.16 3.66

3 Cl -0.040 2.05 3.55

4 O -0.559 1.72 3.22

5 O -0.584 1.72 3.22

6 N -0.074 1.83 3.33

7 N -0.098 1.83 3.33

8 N -0.607 1.83 3.33

9 N -0.893 1.83 3.33

10 C 0.437 2.00 3.50

11 C -1.404 2.00 3.50

12 C -0.315 2.00 3.50

13 C -0.215 2.00 3.50

14 C -0.083 2.00 3.50

15 C -0.192 2.00 3.50

16 C 0.511 2.00 3.50

17 C 0.724 2.00 3.50

18 C 0.902 2.00 3.50

19 C -0.683 2.00 3.50

20 C 0.558 2.00 3.50

21 C -0.280 2.00 3.50

22 C -0.316 2.00 3.50

23 C -0.149 2.00 3.50

24 C -0.266 2.00 3.50

25 C -0.135 2.00 3.50

26 C -0.114 2.00 3.50

27 C -0.049 2.00 3.50

28 C -0.173 2.00 3.50

29 C -0.347 2.00 3.50

30 C -0.121 2.00 3.50

31 C 0.194 2.00 3.50

32 C 0.072 2.00 3.50

33 C -0.104 2.00 3.50

34 C 0.626 2.00 3.50

35 C 0.360 2.00 3.50

36 C -0.438 2.00 3.50

37 H 0.065 1.30 2.80

38 H 0.371 1.30 2.80

39 H 0.099 1.30 2.80

40 H 0.079 1.30 2.80

41 H 0.111 1.30 2.80

42 H 0.100 1.30 2.80

43 H 0.110 1.30 2.80

44 H 0.077 1.30 2.80

45 H 0.086 1.30 2.80

46 H 0.142 1.30 2.80

47 H 0.009 1.30 2.80

48 H 0.166 1.30 2.80

49 H 0.142 1.30 2.80

50 H 0.086 1.30 2.80

51 H 0.086 1.30 2.80

52 H 0.093 1.30 2.80

53 H 0.126 1.30 2.80

54 H 0.075 1.30 2.80

55 H 0.111 1.30 2.80

56 H 0.121 1.30 2.80

57 H 0.067 1.30 2.80

58 H 0.077 1.30 2.80

59 H 0.051 1.30 2.80

60 H 0.082 1.30 2.80

61 H 0.119 1.30 2.80

62 H 0.138 1.30 2.80

63 H 0.068 1.30 2.80

64 H 0.084 1.30 2.80

65 H 0.057 1.30 2.80

66 H 0.417 1.30 2.80

67 H 0.351 1.30 2.80

======================================
